# 我如何开展CD19 CAR-T细胞治疗复发/难治急性B淋巴细胞白血病患者的全程管理

**DOI:** 10.3760/cma.j.cn121090-20250910-00419

**Published:** 2025-12

**Authors:** 迎 王

**Affiliations:** 1 中国医学科学院血液病医院（中国医学科学院血液学研究所），血液与健康全国重点实验室，国家血液系统疾病临床医学研究中心，细胞生态海河实验室，天津 300020 State Key Laboratory of Experimental Hematology, National Clinical Research Center for Blood Diseases, Haihe Laboratory of Cell Ecosystem, Institute of Hematology & Blood Diseases Hospital, Chinese Academy of Medical Sciences & Peking Union Medical College, Tianjin 300020, China; 2 天津医学健康研究院，天津 301600 Tianjin Institutes of Health Science, Tianjin 301600, China

## Abstract

急性B淋巴细胞白血病（B-ALL）目前治疗缓解率高，但复发和难治疾病仍影响长期生存，尤其是在成人患者中。嵌合抗原受体T细胞（CAR-T细胞）疗法是复发/难治B-ALL患者的重要挽救治疗手段，明显提升了这类患者的再缓解率、缓解质量及生存。CAR-T细胞治疗与传统化疗、分子靶向药、抗体药物等的作用机制、不良反应有所不同，尽管有较高的缓解率，但同时也存在独特且部分情况下较为严重的不良反应，加强CAR-T细胞治疗过程的全程管理有助于患者以更好的安全性获得更好的疗效。本文以本中心收治的1例复发/难治B-ALL患者为例，介绍了CD19 CAR-T细胞治疗复发/难治B-ALL时包括患者选择、桥接治疗、清淋化疗、不良反应处理等环节的全流程管理策略。

## Abstract (en)

Remission rate of newly diagnosed B-cell acute lymphoblastic leukemia（B-ALL）based on chemotherapy was high; however, recurrence and refractory diseases still affect long-term survival, especially in adult patients. Chimeric antigen receptor T-cell（CAR-T cell）therapy is an important salvage treatment for patients with relapsed/refractory B-ALL, and it significantly improves the response rate, response quality, and survival of such patients. The mechanism and adverse reactions of CAR-T cellular therapy differed from those of traditional chemotherapy, molecular targeted drugs, antibody drugs, etc. Further, it has unique and, in some cases, more serious adverse reactions, despite its relatively high remission rate. Strengthening the full-process management of CAR-T cellular therapy helps achieve higher efficacy with better safety. The article takes one patient with relapsed/refractory B-ALL treated with CD19 CAR-T at our center as an example, and introduces the full-process management strategy of CAR-T cellular therapy, including patient selection, bridging therapy, lymphodepletion treatment, and adverse reaction management.

急性B淋巴细胞白血病（B-ALL）是常见的急性白血病类型之一，好发于2～5岁儿童和50岁以上中老年人[1]–[3]。基于化疗的诱导治疗方案在儿童和成人B-ALL中能够取得接近90％的完全缓解（CR）率，但复发和难治疾病仍是影响长期生存的棘手问题，尤其是在成人患者中，长期无病生存率只有40％～50％[2]–[3]。嵌合抗原受体T细胞（CAR-T细胞）疗法是复发/难治B-ALL（R/R B-ALL）患者的重要挽救治疗手段。CD19是B淋巴细胞发育过程中全程表达的表面抗原，从幼稚阶段至成熟阶段的B细胞恶性肿瘤均有CD19表达，B-ALL的CD19表达占90％以上，使其成为免疫治疗的理想靶点。目前，已有多款靶向CD19的CAR-T细胞产品在中国、美国等国家上市，用于治疗R/R B-ALL患者[4]–[7]。CAR-T细胞治疗与传统化疗、分子靶向药物、抗体药物等的作用机制、不良反应有所不同，尽管有较高的缓解率，但同时也存在独特的、部分情况下较为严重的不良反应，加强CAR-T细胞治疗过程的全程管理有助于患者更安全的同时获得更好的疗效。本文主要介绍了CD19 CAR-T细胞治疗R/R B-ALL时包括患者选择、桥接治疗、清淋化疗、不良反应处理等环节的管理策略及相关研究进展。

一、典型病例

男，49岁，因咳嗽伴低热1个月余入当地医院。查血常规：WBC 183.04×10^9^/L，HGB 93 g/L，PLT 31×10^9^/L。骨髓形态学考虑ALL。融合基因检测示KMT2A::AF4定量103.62％；染色体核型为46，XY，t（4;11）（q21;q23.3），i（7）（q10），der（17）[1]/46,XY[10]。确诊为B-ALL伴KMT2A重排（既往称为MLL），予VDCP方案化疗1个疗程（4周）后骨髓形态学提示原始淋巴细胞占1.5％，达到血液学CR，后续给予CAM方案巩固化疗1个疗程，骨髓抑制期出现发热，胸部CT提示实变病灶伴晕征，半乳甘露聚糖试验（GM试验）阳性，临床诊断侵袭性曲霉菌病，给予伏立康唑输注后体温控制，白细胞恢复后复查骨髓提示血液学复发，遂就诊我院。入院后复查胸部CT病灶较前略有缩小，继续给予伏立康唑输注，骨髓细胞形态学示原始淋巴细胞占63.5％，流式细胞术免疫表型示异常B淋巴母细胞占有核细胞64.46％，表达CD38、CD19，部分表达CD81、CD20、CD34，弱表达CD22、CD45，不表达CD10，KMT2A::AF4融合基因定量96.25％。患者化疗后早期复发，但合并肺部侵袭性曲霉菌病，无法入组临床试验，需从疗效及安全性角度综合考虑为患者选择下一步治疗方案。R/R B-ALL目前可用的治疗包括贝林妥欧单抗（CD3-CD19双特异性抗体）、奥加伊妥珠单抗（CD22单抗链接刺孢霉素的抗体药物偶联物）、CAR-T细胞治疗以及传统化疗。患者高白细胞起病，伴有KMT2A重排，为高危患者，标准化疗达血液学CR后短期出现复发，再次采用化疗获得缓解的概率较低，免疫治疗对于R/R B-ALL患者疗效优于传统化疗，而免疫治疗中，CD19 CAR-T细胞治疗相较于抗体治疗的缓解率及可检测残留病（MRD）转阴率均更高，因此选择CD19 CAR-T细胞治疗作为患者的挽救治疗。遂采集患者自体外周血单个核细胞（PBMC）制备以4-1BB为共刺激域的CD19 CAR-T细胞（antiCD19scFv-4-1BB-CD3ζ），由于细胞制备及质控放行约需要1个月时间，且高肿瘤负荷患者较低肿瘤负荷患者在接受CD19 CAR-T细胞治疗过程中更容易发生严重细胞因子释放综合征（CRS）和（或）免疫效应细胞相关的神经毒性综合征（ICANS），因此在等待CAR-T细胞制备期间，通常对于高肿瘤负荷患者采用桥接治疗。理想的桥接治疗能有效降低肿瘤负荷，同时安全性良好，尤其是血液学不良反应轻，以避免由于桥接治疗骨髓抑制重，出现感染等并发症或已有感染进一步加重影响后续CAR-T细胞回输，抗体治疗在疗效及血液学不良反应上均优于化疗。另外，由于CD19 CAR-T细胞治疗缓解后有部分患者因为抗原免疫逃逸导致CD19阴性复发，因此为实现不同靶点的免疫治疗药物双靶点打击，选择靶向CD22的奥加伊妥珠单抗在CD19 CAR-T细胞制备期间作为桥接治疗，同时兼顾降低CAR-T细胞回输前肿瘤负荷及双靶点打击。该患者接受奥加伊妥珠单抗（1 mg/d，第1、8、15天）作为桥接治疗后复查骨髓流式细胞术MRD为2.20％，CD19阳性，KMT2A::AF4融合基因定量：12.47％。桥接治疗期间持续伏立康唑抗感染治疗，肺部感染灶较前进一步缩小。遂开始CD19 CAR-T细胞治疗，行氟达拉滨（Flu）与环磷酰胺（Cy）的组合（Flu/Cy）预处理后回输0.5×10^8^个靶向CD19的CAR阳性T细胞。于回输后第7天出现发热，38.9 °C，生命体征平稳，考虑1级CRS，给予布洛芬对症退热后好转，CRS持续1 d，无ICANS发生。回输后首月每周流式细胞术检测外周血CAR-T细胞扩增情况，峰值发生于回输后第14天，CAR-T细胞计数为157个/µl，同时第14天时检测外周血CD19^+^B淋巴细胞计数为0。回输后第28天复查骨髓达CR，流式细胞术MRD阴性，KMT2A::AF4融合基因定量：0％，NGS-IgH重排阴性，达到深度缓解。CAR-T细胞治疗过程中持续伏立康唑抗感染治疗，过程平稳。关于R/R B-ALL患者接受CD19 CAR-T细胞治疗达到缓解后是否需要桥接异基因造血干细胞移植（allo-HSCT）巩固尚无统一建议，目前已知复发风险相对较高的因素包括既往多线治疗、合并髓外疾病、高危白血病细胞遗传学改变（如TP53改变、KMT2A重排、Ph like、IKZF改变等）。另外，KMT2A重排是发生谱系转换的高危因素。鉴于患者高白细胞发病，伴KMT2A重排，考虑存在再次复发风险，且患者有合适的供者，肺感染较前明显好转，CAR-T细胞回输后2个月复查外周血CAR-T细胞计数为6个/µl，CD19^+^ B淋巴细胞计数仍为0。遂于CAR-T细胞回输后2个月余行单倍体allo-HSCT（女供父），现移植后1年半余，无病生存。

二、患者选择

基于中华医学会血液学分会、中国医师协会血液科医师分会和中国临床肿瘤学会（CSCO）白血病专家委员会以及欧洲血液和骨髓移植协会（EBMT）、国际细胞治疗学会（ISCT）与EBMT联合认证委员会（JACIE）、欧洲血液学协会（EHA）的临床实践建议[8]–[9]，同时整合关键临床试验入排标准[4]–[7]和真实世界研究中的疗效/安全性数据[10]–[11]，我们归纳了R/R B-ALL患者真实世界接受CD19 CAR-T细胞治疗的筛选标准：

（1）年龄：无限制，尽管多数临床研究限制了入组患者的年龄，但真实世界研究数据显示老年患者疗效与安全性与成年患者相似。

（2）体能状态：多数临床研究限制美国东部肿瘤协作组（ECOG）评分≤1分，ECOG>1分的患者与不良预后相关，真实临床实践中会有相当一部分患者ECOG>1分，在进行CAR-T细胞治疗前应与患者及家属充分沟通风险与获益，并做好全程管理。

（3）肿瘤负荷：无绝对限制，但基线高肿瘤负荷与CAR-T细胞治疗不良反应风险增加及长期生存率下降相关。

（4）其他恶性肿瘤：合并其他活跃恶性肿瘤的患者建议暂缓CAR-T细胞治疗，需权衡风险与收益。

（5）既往治疗：allo-HSCT患者需无移植物抗宿主病证据且停用免疫抑制剂至少1个月，其余无限制，不过既往allo-HSCT、贝林妥欧单抗、奥加伊妥珠单抗治疗失败或复发的患者，其CAR-T疗效略差。

（6）单采前治疗：单采前细胞毒性化疗药物和糖皮质激素可影响T细胞功能，需要一定时间洗脱药物。单采前糖皮质激素和短效细胞毒性药物至少停药3 d，高剂量化疗则需要3～4周。

（7）感染：活动性细菌、真菌感染，病毒血症为禁忌证。潜伏性病毒感染须接受预防性抗病毒治疗，如乙肝表面抗原阳性，即使HBV-DNA阴性，也应持续口服恩替卡韦预防HBV再激活。

（8）中枢神经系统疾病：多数临床研究排除了活动性中枢神经系统白血病的患者，但真实世界研究和部分研究者发起临床研究显示这些患者也可从CAR-T细胞治疗中获益。

（9）其他检查：心脏超声评估心脏功能，建议左室射血分数>40％；心电图排除严重心律失常；肝肾功能建议胆红素<34 µmol/L，AST、ALT<4倍正常范围上限，肌酐清除率>30 ml/min；血常规：PLT>30×10^9^/L，绝对淋巴细胞计数>0.2×10^9^/L。

三、输注前桥接治疗与清淋预处理

在PBMC采集与CAR-T细胞输注两个时间点之间有3～4周的细胞制备期，在此期间可选用适当药物进行桥接治疗。桥接治疗的目的是降低肿瘤负荷，避免患者病情在等待期内进展，同时应注意避免桥接治疗相关的不良反应。如本例患者，我们选择不良反应小、血液学毒性轻、且靶向不同靶点（CD22）的抗体药物偶联物奥加伊妥珠单抗作为桥接治疗，在细胞制备的等待期间兼顾了降低肿瘤负荷和安全性。另外，也可选择单个周期低强度化疗方案（长春碱类、糖皮质激素、巯嘌呤等），并根据疾病负荷、既往治疗情况、既往不良反应等情况进行个体化调整。Ph^+^ALL可以选择酪氨酸激酶抑制剂（TKI）联合或不联合低强度化疗作为桥接治疗[12]。中枢神经系统白血病的患者可在CAR-T细胞回输前等待期通过腰椎穿刺+鞘内注射减少中枢神经系统中的肿瘤负荷，有明确占位的中枢神经系统侵犯或者其他髓外侵犯的患者也可考虑放疗联合小剂量化疗作为桥接治疗。

清淋是在输注CAR-T细胞前1周内通过化疗药物清除患者体内淋巴细胞，为后续CAR-T细胞在体内扩增和持久存活创造有利条件的关键步骤。清淋的优势包括降低肿瘤负荷、清除内源性淋巴细胞、促进稳态细胞因子产生、改善肿瘤微环境和减弱抗CAR免疫反应等。目前常用的方案为Flu/Cy，常用剂量见表1[13]。本中心通常使用Flu 25～30 mg·m^−2^·d^−1^，−5～−3 d；Cy 300 mg·m^−2^·d^−1^，−5～−3 d。有研究发现在Flu/Cy方案中加入表观遗传调节剂地西他滨可改善B-ALL患者长期生存[14]，适用于何种患者尚需探索。

**表1:** 嵌合抗原受体T细胞（CAR-T细胞）治疗复发/难治急性B淋巴细胞白血病患者前的清淋方案

临床试验注册号	氟达拉滨	环磷酰胺
NCT05727683	25 mg·m^−2^·d^−1^，−4、−3、−2 d	250 mg·m^−2^·d^−1^，−4、−3、−2 d
NCT04206943	30 mg·m^−2^·d^−1^，−5、−4、−3 d	300 mg·m^−2^·d^−1^，−6 d
NCT03321123	30 mg·m^−2^·d^−1^，−5、−4、−3、−2 d	500 mg·m^−2^·d^−1^，−5、−4 d
NCT02614066	25 mg·m^−2^·d^−1^，−4、−3、−2 d	900 mg·m^−2^·d^−1^，−2 d
NCT04684147	30 mg·m^−2^·d^−1^，−5、−4、−3、−2 d	500 mg·m^−2^·d^−1^，−5、−4 d
ChiCTR-OPN-16008526	25 mg·m^−2^·d^−1^，−4、−3、−2 d	300 mg·m^−2^·d^−1^，−4、−3、−2 d
ChiCTR-OIC-17013523	30 mg·m^−2^·d^−1^，−5、−4、−3 d	250 mg·m^−2^·d^−1^，−5、−4、−3 d

四、不良反应管理

1. CRS：CRS是CAR-T治疗过程中常见的急性毒性反应，首发症状常为发热，其他症状包括窦性心动过速、低血压、呼吸困难、低氧血症等。国内国际常用的CRS分级方式为ASTCT标准[15]，并根据CRS分级指导治疗。国内外有多项专家共识指导CAR-T细胞相关毒性的处理，对于1级CRS只有发热症状（≥38 °C），通常建议只需要对症处理并排除感染；对于发热持续>3 d或出现2级CRS，此时需要启用托珠单抗。托珠单抗是一种针对IL-6受体的人源单克隆抗体，推荐剂量为静脉注射8 mg/kg，若症状无改善可在8 h后重复使用，累计使用次数不应超过4次。使用1～2剂托珠单抗后症状无改善的2级CRS需静脉注射地塞米松10 mg每12 h 1次；地塞米松使用24 h后仍无效的2级CRS或3级CRS需增加地塞米松剂量至10～20 mg，每6 h 1次；对于危及生命的4级CRS应使用静脉大剂量甲泼尼龙，具体剂量为500 mg每12 h 1次，连续3 d，随后250 mg每12 h 1次连续2 d，125 mg每12 h 1次连续2 d，最后60 mg每12 h 1次直到CRS缓解至1级。糖皮质激素在CRS缓解后应尽快减量。对于上述标准治疗仍难治的CRS可尝试使用IL-1受体抑制剂阿那白滞素、JAK抑制剂芦可替尼和血浆置换等疗法[16]。对于低氧血症和低血压应根据严重程度采用不同的支持治疗，如使用升压药物、高流量给氧或机械通气[17]。鉴于R/R B-ALL接受CAR-T细胞治疗后有可能发生高级别CRS，尤其是肿瘤负荷高的患者，因此本中心对于R/R B-ALL患者CRS的处理经验是早干预，通常在发生1级CRS时就启动托珠单抗或激素治疗，曾有报道早期使用糖皮质激素并未影响CAR-T细胞扩增[18]–[19]，我们的临床实践也支持这一观点。

2. ICANS：ICANS常见症状为失语、意识水平改变、认知障碍、运动无力、癫痫发作及脑水肿。根据ASTCT共识，ICANS的分级根据免疫效应细胞相关脑病评分（ICE）、意识水平、癫痫发作、运动障碍和颅内压升高/脑水肿五项中最严重的事件来确定[15]。ICANS的治疗更加强调糖皮质激素，尤其是透过血脑屏障效果相对更好的地塞米松（剂量同CRS处理），ICANS的发生通常晚于CRS，除非ICANS患者同时合并CRS，托珠单抗一般不用于ICANS，发生危及生命的4级ICANS则需要静脉注射甲泼尼龙1 000 mg/d，持续3 d。糖皮质激素应持续使用直到ICANS缓解至1级，随后快速减量停药[17],[20]。辅助检查如MRI、EEG、眼底和脑脊液检查有助于评估神经毒性并鉴别诊断，尤其是R/R B-ALL患者CAR-T细胞治疗后可能存在严重粒细胞缺乏（粒缺）、免疫功能缺陷，患者出现神经症状时需鉴别ICANS和中枢神经系统感染，抑或两者同时存在，另外，还有脑血管意外等需要鉴别。其他对症支持治疗如镇静药物、降颅压药物、抗癫痫药物应根据患者病情考虑使用，≥3级ICANS患者应转入ICU。对于难治的ICANS可尝试难治性CRS中使用的抗细胞因子疗法，也可尝试鞘内化疗或鞘内注射激素治疗[21]。曾有报道一例R/R B-ALL患者接受CD19 CAR-T细胞后发生严重难治ICANS，经地塞米松、大剂量甲泼尼龙冲击治疗无效，环磷酰胺1.5 g/m^2^输注后成功治疗，考虑环磷酰胺通过迅速清除CAR-T细胞起效[22]。本中心对于ICANS的处理经验是勤观察、早发现、早干预，对于R/R B-ALL患者接受CAR-T细胞回输后每日早晚两次进行ICE评分，考虑ICANS即启动地塞米松及其他对症支持治疗，神经内科会诊、HCU/ICU及时转诊也非常重要。

3. 免疫效应细胞相关噬血细胞性淋巴组织细胞增生症（IEC-HS）：IEC-HS为CAR-T细胞治疗相关的继发性噬血细胞性淋巴组织细胞增生症（HLH），以过度免疫激活为特征，一般发生晚于CRS，可导致严重多器官功能障碍甚至死亡[23]。其严重程度评估依据ASTCT发布的共识[23]。目前推荐的IEC-HS一线治疗为阿那白滞素±糖皮质激素，在发生2级IEC-HS时开始干预。阿那白滞素剂量为100～200 mg，每6～12 h 1次，地塞米松剂量同CRS。若48 h仍未缓解，则联用阿那白滞素和糖皮质激素并增加药物剂量，并可考虑加用二线治疗芦可替尼，本中心通常使用10 mg口服每12 h 1次[23]。对于二线治疗仍难治的IEC-HS可使用γ干扰素单克隆抗体（依马利尤）或依托泊苷等[23]–[24]。

4. 免疫效应细胞相关血液毒性（ICAHT）：ICAHT指CAR-T细胞治疗后出现的血细胞减少症，分为早期ICAHT（回输后30 d内）和晚期ICAHT（回输后30 d后）[25]。目前对ICAHT的治疗尚无前瞻性研究数据支持，通常以成分输血支持和促进造血药物为主。粒细胞集落刺激因子（G-CSF）、重组人促血小板生成素（rhTPO）或TPO受体激动剂是促进造血常用的药物。粒-巨噬细胞集落刺激因子（GM-CSF）刺激单核巨噬细胞活化，促进加重CRS，因此GM-CSF应避免使用；对于G-CSF的使用时机目前存在争议，本中心通常在CRS、ICANS和（或）IEC-HS控制后启动G-CSF治疗[25]–[26]。

5. 感染：感染在输注CAR-T的R/R B-ALL患者中相对常见，因为这类患者通常有严重且持续时间相对长的粒缺期。CAR-T细胞输注前建议筛查HIV、HBV、HCV的感染指标，其他病原体可根据患者风险因素纳入筛查。CAR-T细胞输注后30 d内细菌、真菌感染风险相对高，本中心对于粒缺患者通常予以口服可以覆盖曲霉菌、毛霉菌的泊沙康唑进行真菌预防，至中性粒细胞恢复至0.5×10^9^/L以上；复方磺胺甲噁唑则建议CAR-T细胞输注后常规使用，以预防肺孢子菌感染，从回输前1周直至CD4^+^淋巴细胞计数>200个/µl。目前尚无循证医学证据支持在CAR-T细胞治疗的同时给予常规细菌预防。CAR-T细胞输注后病毒感染不容忽视，尤其30 d后，抗病毒药物阿昔洛韦或伐昔洛韦应从清淋治疗开始时使用，直到CAR-T细胞输注后至少6个月。对于HBsAg监测阳性的患者，应从清淋治疗开始使用抗乙肝病毒药物预防，本中心通常使用恩替卡韦0.5 mg/d，贯穿CAR-T细胞治疗全程管理，监测患者外周血淋巴细胞亚群，当出现外周血B淋巴细胞恢复后继续预防性使用12个月，以防止HBV再激活。停止预防后也还需要定期监测HBV DNA、HBV血清学标志物和肝功能，以便及时发现并应对HBV再激活，半年内每月监测1次，半年后每3个月监测1次，直至停用抗乙肝病毒药物后12个月，具体参见中国专家共识[27]。

6.低丙种球蛋白血症：低丙种球蛋白血症也是CAR-T细胞治疗后常见的并发症，血清IgG<4 g/L或血清IgG在4～6 g/L且合并严重/反复感染的患者，给予静脉补充丙种球蛋白。对于CAR-T细胞回输后早期出现发热的患者应进行病原学检查，以鉴别感染与CRS，避免贻误治疗[28]–[29]。

五、巩固及维持治疗

关于R/R B-ALL患者接受CD19 CAR-T细胞治疗达到缓解后是否需要桥接allo-HSCT巩固尚存在争议，其对R/R B-ALL患者长期生存的影响在不同研究中结论不一：有许多回顾性研究认为巩固治疗采用allo-HSCT显著改善了R/R B-ALL患者的无白血病生存期和总生存期[30]–[32]，然而也有研究并没有观察到桥接移植患者相对于非移植患者的生存优势[33]–[34]。患者是否能从allo-HSCT中获益可能取决于自身的复发风险，包括既往多线治疗、合并髓外疾病、高危白血病细胞遗传学特征如TP53改变、KMT2A重排、Ph like、IKZF改变等均具有较高复发风险[35]。另外，动态监测B细胞早期恢复和MRD水平变化有助于复发的预测。如条件允许，对于高复发风险的R/R B-ALL患者推荐在CAR-T细胞治疗缓解后接受allo-HSCT。移植时机方面，本中心通常选择CAR-T细胞回输后2～3个月，主要有以下考虑：此时绝大部分患者已经从免疫治疗早期相关不良反应中恢复，对移植相关并发症的影响相对小；既往研究显示CAR-T细胞在体内可检测到的中位时间是92 d[7]，3个月后复发风险可能增加。

R/R B-ALL患者接受CD19 CAR-T治疗达到缓解后，不采用allo-HSCT巩固的患者是否需要维持治疗？采用何种维持治疗？目前仅Ph^+^ALL，各中心均采用TKI为基础的维持治疗；而对于Ph^-^ALL，目前有很多中心开展不同临床研究探索维持治疗，包括序贯CD22 CAR-T、CD22抗体、地西他滨、西达本胺、维奈克拉、来那度胺、小剂量化疗、肿瘤疫苗等，但目前尚缺乏循证医学证据。

六、挽救治疗

尽管CD19 CAR-T细胞在R/R B-ALL患者中取得了较高缓解率，但仍有50％左右的患者在CAR-T细胞输注后复发[36]。对于CAR-T细胞后复发的患者首先建议参加临床试验，如不能入组临床试验，鉴于R/R B-ALL在CAR-T细胞输注前大多接受了多线化疗，化疗使这些患者再度缓解的机会不大，因此应将挽救治疗的选择关注于免疫治疗和靶向药物。在挽救治疗前应当根据复发时白血病细胞上抗原的表达情况选择后续治疗。如果患者复发时保留CD19表达，则可以使用靶向CD19的药物（如CD19 CAR-T细胞二次输注、贝林妥欧单抗）[37]，如CD22表达也可以使用靶向其的药物（如奥加伊妥珠单抗，参加CD22 CAR-T细胞临床试验）；如果复发时CD19、CD22均不表达，可考虑分子靶向药物联合化疗或其他[38]。谱系转换是一种特殊类型的复发模式，复发时淋系抗原的丢失使上述策略难以取得疗效，此时使用髓系肿瘤治疗方案可能有效[39]–[40]。上述挽救治疗取得再次缓解的患者应接受allo-HSCT以巩固疗效。

七、结语

白血病的治疗需要制定整体治疗策略、做好全程管理，一系列新药的问世不断地提升白血病患者的疗效，如何把各种治疗手段用好是医师的持续关注点，兼顾疗效、安全性、可及性打好治疗的“组合拳”是医师持续努力的方向。
